# Promoter Hypermethylation and Decreased Expression of Syncytin-1 in Pancreatic Adenocarcinomas

**DOI:** 10.1371/journal.pone.0134412

**Published:** 2015-07-31

**Authors:** Qinsheng Lu, Jinping Li, Christopher Senkowski, Zuoqing Tang, Jianhao Wang, Tianhe Huang, Xue Wang, Karen Terry, Steven Brower, Wayne Glasgow, Haibin Chen, Shi-Wen Jiang

**Affiliations:** 1 Department of Histology and Embryology, Shantou University Medical College, Shantou, Guangdong, China; 2 Department of Biomedical Science, Mercer University School of Medicine, Savannah, GA, United States of America; 3 Department of Surgery, Curtis and Elizabeth Anderson Cancer Institute, Memorial Health University Medical Center, Savannah, GA, United States of America; 4 Department of Medical Genetics, School of Basic Medical Sciences, Capital Medical University, Beijing, China; 5 School of Pharmaceutical Engineering and Life Science, Changzhou University, Changzhou, Jiangsu Province, China; 6 Department of Surgery & Surgical Oncology, Beth Israel Medical Center, New York, NY, United States of America; 7 Department of Obstetrics and Gynecology, the Second Affiliated Hospital of Wenzhou Medical University, Wenzhou 325027, China; 8 Department of Obstetrics and Gynecology, Memorial Health University Medical Center, Savannah, GA, United States of America; Peking University Health Science Center, CHINA

## Abstract

Syncytin-1 is a member of human endogenous retroviral W gene family (*HERVW1*). Known to be expressed in human placental trophoblast, syncytin-1 protein mediates the fusion of cytotrophoblasts for the formation of syncytiotrophoblasts, the terminally differentiated form of trophoblast lineage. In addition, *in vitro* studies indicate that syncytin-1 possessed nonfusogenic functions such as those for immune suppression, cell cycle regulation and anti-apoptotic activities. Overexpression of syncytin-1 has been observed in various malignant tissues including breast, endometrial and ovarian cancers. It was reported that syncytin-1 gene expression is associated with dynamic changes of DNA hypomethylation in the 5’ LTR. In this study, applying the real-time PCR, Western blot analysis and immunohistochemistry methods, we demonstrate a constitutive expression of syncytin-1 in normal pancreas tissues as well as normal tissues adjacent to cancer lesions. Moreover, a reduced expression is found in the pancreatic adenocarcinoma tissues. The expression levels of syncytin-1 are not correlated with the stage, historical grade and gender, but inversely correlated with patients’ age. Furthermore, COBRA and bisulfite sequencing results indicated that the lower expression of syncytin-1 is correlated with the hypermethylation of two CpG dinucleotides in the 5’ LTR of syncytin-1 gene. The nonfusogenic function of syncytin-1 in normal pancreas as well as its role(s) in the pathogenesis and progression of pancreatic cancers remains to be investigated. Identification of the two CpG dinucleotides around transcription start site as key epigenetic elements has provided valuable information for further studies on the epigenetic regulation of syncytin-1 in pancreatic cancer cells.

## Introduction

Ranked as the fourth most deadly cancer for both females and males in the United States, pancreatic cancer patients have a 5-year overall survival rate lower than 6% [1, 2]. It was estimated that 46,420 new cases were diagnosed for this disease in 2014, which caused 39,590 deaths in the United States along [1]. Worldwide, pancreatic cancers accounted for 4% of estimated new cancer deaths in 2012 [3]. Multiple genetic and epigenetic alterations have been identified in pancreatic cancers, but the precise pathological mechanisms remain poorly understood. The lack of knowledge in this area has impeded the development of advanced diagnostic and treatment modalities for more effective management of this deadly disease.

Numerous studies have focused on the genetic alterations and their involvement in pancreatic cancers, and multiple familial and somatic mutations were found to be contributing factors (reviewed in [4]). Mutations of *KRAS* oncogene and tumor suppressor genes such as *p16*, *p53*, *DPC4*, *BRCA2*, *LKB1*, and *MKK4* were detected in pancreatic cancers at varied frequencies (reviewed in [2]). Individuals with hereditary syndromes such as hereditary breast cancer, FAMMM, Peutz-Jeghers, Fanconi anemia, cystic fibrosis, and ataxia telangiectasia have shown an increased risk of pancreatic cancer [2, 4]. In addition, recent findings on epigenetic events and mechanisms have much enriched our knowledge on the pathogenesis of pancreatic cancer. Changes of DNA methylation pattern, with their direct implication for gene expression and genetic mutations, have been recognized as important tumorigenic pathways [4–7]. DNA hypomethylation, which usually associates with gene activation during the carcinogenesis of pancreatic cancer, was observed in oncogenes such as *S100A4*, *CLDN4*, *LCN2*, *SFN*, *TFF2*, *MSLN*, and *PSCA* [8]. In contrast, aberrant hypermethylation has been detected in tumor suppressor genes including *PTEN*, *CDKN2A*, *and RASSF1A* in pancreatic cancers [8]. DNA hypermethylation was also identified in precancerous lesions such as mucinous cystic neoplasms (MCNs), intraductal papillary mucinous neoplasms (IPMNs) and pancreatic intraepithelial neoplasia (PanIN) [8–10], pointing to an early involvement of epigenetic alterations in the development of pancreatic cancers.

Syncytin-1 is encoded by human endogenous retroviral envelope protein gene (*HERVW1*) located to chromosome 7 (7q21.2) [11–13]. In human, syncytin-1 is most abundantly expressed in the placental trophoblast lineage. Syncytin-1 has been known for its important role in the terminal differentiation of trophoblast lineage by mediating the formation of syncytiotrophoblasts through its fusogenic activity [11, 12, 14, 15]. However, recent data from this and other laboratories provided strong support for a nonfusogenic activity of syncytin-1 [16–22]. Syncytin-1 appears to exert a regulatory activity in the homeostasis of placental trophoblast lineage [11, 15, 23]. Syncytin-1 promotes the proliferation of cytotrophoblasts, the building blocks of syncytiotrophoblasts or syncytium [22]. Moreover, syncytin-1 has shown anti-apoptotic activity in both knockdown and overexpression models *in vitro* [20, 24]. It was proposed that through regulation of cytotrophoblast growth, as the input, and cell fusion as well as cell apoptosis, as the output, of the trophoblast pool, this single factor may constantly modulate the trophoblast lineage development during placental maturation [20, 22].

It is noteworthy that recent studies indicated that syncytin-1 expression is activated and upregulated in a variety of malignancies including breast cancer [16, 25], endometrial carcinomas [17, 18, 26], ovarian cancer [27], colorectal cancer [19], leukemia and lymphoma [28]. Although syncytin-1 levels appear to be related to clinical manifestation of cancer patients, the pathological significance of its nonfusogenic activities remains to be investigated. Several studies suggested that measurement of syncytin-1 expression levels in cancer tissues may carry some prognostic values for certain tumor types and stages [18, 19, 25].

Accumulated data indicated that the specific syncytin-1 expression in placental tissues is predominantly controlled by epigenetic mechanism [18, 27, 29–32]. High methylation levels of the 5’ LTR of syncytin-1 gene are invariably observed in tissues without syncytin-1 expression. In placental trophoblasts, the 5’ LTR becomes hypomethylated, and the levels of expression are negatively correlated with the DNA methylation levels [29, 30]. Compared to human placenta, syncytin-1 regulation in cancer cells is poorly characterized. Okahara et al. showed that families of human endogenous retroviruses including HERV-H13, HERV-Fb1, HERV-K (HML6-1) and HERV-K (HML6-c14), were not expressed in human pancreas [33]. Results from Yi et al. demonstrated that all the structural genes (*gag*, *pol*, *env*) of HERV-W family were expressed in the pancreas of Japanese monkey (*Macaca fuscata*) as assessed by by RT-PCR and sequencing analyses [34]. However, in year 2000 Mi et al. reported that syncytin-1 mRNA expression was undetectable in human pancreas when Northern hybridization [11], an outdated technique with relatively low sensitivity and accuracy, was applied. Thus, the expression pattern and potential biological function of syncytin-1 in normal and malignant pancreatic tissues remain unclear.

In the current study, we measure the mRNA and protein expression levels of syncytin-1 in pancreatic cancers and analyze the relationship between syncytin-1 expression and clinical parameters. Moreover, we determine the DNA methylation patterns in the 5’ LTR of syncytin-1 and how the methylation levels may affect its expression. The findings have paved the road for in-depth studies on the role(s) of syncytin-1 in pancreatic normal physiology and malignant transformation.

## Materials and Methods

### Ethics statement

This study was reviewed and approved by the Internal Review Board of Memorial Health University Medical Center. Written informed consent was obtained from each study subject.

### RNA isolation, cDNA synthesis, and quantitative real-time PCR

Pancreatic adenocarcinoma tissues (n = 30) and normal pancreatic tissues adjacent to cancer lesions (n = 10) were dissected following surgery. Specimens were thoroughly rinsed with PBS and stored at −80°C for RNA, DNA and protein isolation.

Total RNA was isolated from tissue samples with the use of RNeasy Plus Mini Kit (Qiagen, Valencia, CA, USA) according to manufacturer’s recommendation. RNA was quantified using Nanodrop-1000 spectrophotometer (Thermo Fisher Scientific, Wilmington, DE, USA). cDNA synthesis was performed with High Capacity RNA-to-cDNA Kit (Applied Biosystems, CA, USA), using 1 μg of RNA in 20 μL reactions. cDNA in 20 μL original volume was diluted to 100 μL by addition of 80 μL nuclease-free water. Real-time PCR was performed in 12 μL reactions containing 6 μL of 2x SYBR Green PCR Master Mix (USB Products, Affymetrix, USA), 1 μL of forward primer (10 μM), 1 μL of backward primer (10 μM), 2 μL of nuclease-free water and 2 μL of diluted cDNA solution. The following conditions were used for PCR reaction: initial denaturation at 95°C for 10 min, followed by 40 cycles of denaturation at 95°C for 15 s, and annealing/extension at 60°C for 1 min. Reactions were carried out on the ABI 7900HT Fast Real-Time PCR System (Applied Biosystems, CA, USA). PCR primers were: Syncytin-1-forward: 5’-TCATATCTAAGCCCCGCAAC, and Syncytin-1-backward: 5’-CGCCAATGCCAGTACCTAGT, generating a 90 bp amplicon; β-actin-Forward: 5’-CGCGAGAAGATGACCCAGAT, β-actin-backward: 5’-ACAGCCTGGATAGCAACGTA, generating a 71 bp amplicon. PCR products were resolved in 1.8% TAE agarose gels and visualized under UV light following ethidium bromide staining. β-actin was used as internal reference gene. The threshold cycles (Ct) were determined in triplicate for each sample. Results of syncytin-1 were standardized with those from the β-actin internal control gene. The mRNA levels were expressed as relative folds of change over normal controls, in the form of Mean ± SEM.

### Western blot analysis

Proteins were extracted from pancreatic tissues using RIPA buffer (Boston BioProducts, Boston, MA, USA) containing 1 mM phenylmethylsulfonyl fluoride (PMSF), 5 mM sodium fluoride (NaF), and 1 mM sodium vanadate (Na_3_VO_4_), supplemented with Protease Inhibitor Cocktail (100X) (Thermal Scientific, Rockford, IL, USA). Frozen pancreatic tissues were grounded in liquid nitrogen and lysed in 0.5 mL of lysis buffer. Following centrifugation at 14,000 rpm for 30 min at 4°C, the supernatants were collected and stored at - 80 °C in aliquots. Protein concentrations were measured with the BCA method (Thermo Scientific, Rockford, IL, USA). 40 μg of protein extracts were resolved in 12% SDS polyacrylamide gels and transferred to polyvinylidene fluoride (PVDF) membranes. After blocking nonspecific reaction for 2 h at room temperature with TBS containing 0.1% Tween 20 (TBST) and 5% non-fat dry milk, the blots were incubated with rabbit anti-syncytin-1 (1:800, GTX70327, Gene Tex, Irvine, CA, USA) or rabbit anti-GAPDH (1:1000, #5174P, Cell Signaling, Beverly, MA, USA) for 2 h at room temperature in TBST containing 5% non-fat milk. Following three time of washing with TBST, the blots were incubated in TBST containing goat anti-rabbit secondary antibody (1:6000, sc-2301, Santa Cruz Biotechnology, Inc., Dallas, Texas, USA) for 1 h at room temperature. Color development was carried out with ECL substrates (Thermo Scientific, Rockford, IL, USA). Results of GAPDH expression provided controls for protein loading.

### Immunohistochemistry

A pancreas cancer tissue array was purchased from US Biomax Inc. (PA805a, Rockville, MD, USA). This array contains normal pancreas tissues from non-cancer patients, cancer-adjacent normal tissues, and pancreatic adenocarcinoma tissues. For deparaffinization and rehydration, the tissue array was sequentially treated with xylene (20 min x 3), 100% ethanol (5 min x 2), 95% ethanol (5 min x 2), 80% ethanol (5 min x 1), 70% ethanol (5 min x 1) and distilled water (5 min × 2). Immunohistochemistry Accessory Kit (IHC-101, Bethyl Laboratories, Inc., Montgomery, TX, USA) was applied for antigen epitope retrieval, quenching of endogenous peroxidase, slide washing, and secondary antibody incubation following manufacture’s recommendations. After blocking, the slide was incubated overnight at 4°C with the primary antibody, rabbit polyclonal anti-HERV-W (1:50, Cat.#ab71115, Abcam Inc., Cambridge, MA, USA). Following extensive washing, secondary antibody (1:500, IHC-101, Bethyl Laboratories, Inc., Montgomery, TX, USA) was applied. Color development was carried out using diaminobenzidine tetrahydrochloride (DAB, Bethyl Laboratories, Inc., Montgomery, TX, USA). Tissue slides were counterstained with haematoxylin (Santa Cruz Biotechnology, Inc., Dallas, Texas, USA). Slides were dehydrated with gradient alcohol solutions and 100% xylene in the reverse order as applied in the deparaffinization step, and mounted with non-aqueous media before observation under microscope. Images were taken with a Nikon DS-Fi2 camera (Nikon Instruments Inc., New York, USA). Semi-quantitative assessment was performed by scoring the staining intensity as 0, 1, 2, or 3, which corresponded to negative, weak, moderate, and strong staining intensities, respectively.

### DNA extraction and bisulfite modification

Genomic DNA was isolated from pancreas tissues with QIAmp DNA Mini Kit (Qiagen, Valencia, CA, USA). One μg of genomic DNA was applied for bisulfite conversion. The conversion reaction and subsequent purification was carried out with Qiagen EpiTect Bisulfite Kit (Qiagen, Valencia, CA, USA) according to the manufacturer's instructions. Bisulfite-treated DNA was stored at −20°C for later assays. The results of DNA sequencing showing 100% cytosine-to-thymine conversion of cytosines from non-CpG dinucleotide contexts indicated a high conversion rate of unmethylated cytosines.

### Combined bisulfite restriction analysis (COBRA) and bisulfite sequencing

PCR primers previously used in this laboratory were applied for COBRA [35]: syn-1-F: 5’-ATATTTTTTGGAGAGTGAATTATTGAGTTA; syn-1-R: 5’-AAAAACAACTCCCATACAAAAAAAA. The 456 bp PCR amplicon covers 7 CpG sites from the 5’ LTR of syncytin-1 gene on chromosome 7 (7q21.2). The PCR reaction was performed with HotStart Taq Master Mix (QIAGEN, Valencia, CA, USA) in 20 μL reactions containing 10 μL of 2x HotStar Taq Master Mix, 7 μL of nuclease-free water, 0.5 μL of forward and backward primer each (10 μM stock), and 2 μL of bisulfite-treated DNA templates. PCR conditions were: 10 min at 95°C for initial denaturation, followed by 40 cycles of: 30 s at 95°C for denaturation; 30 s at 50.5°C for annealing; 1 min at 72°C for extension; and 10 min at 72°C for the final extension. To increase PCR products, a second-round PCR was performed with 1 μL of first-round PCR products as template, applying the same conditions as in first-round. Six μL of the PCR products were digested using 2 units of restriction enzyme Acl I (cut at AACGTT) (New England BioLabs Inc., Ipswich, MA, USA in a final volume of 20 μL at 37°C for 2 h. Cleavage only occurs if the cytosines in the restriction sites are preserved as cytosines during the bisulfite modification as a result of methylation. Restriction digestion conditions and amounts of enzymes were optimized for a complete digestion of PCR products. The digested PCR products were separated in 2.0% agarose gels (Fermentas, Glen Burnie, MD, USA). Gel pictures were taken with GelDoc-It TS Imaging Systerm (UVP, LLC, Upland, CA, USA) and subjected to densitometry analysis with Image J software (NIH, Bethesda, MD, USA). The methylation index was calculated as a percentage of the density of two cleaved bands (134 bp and 322 bp) that represent methylated syncytin-1, in the total density of DNA fragments including both cleaved (134 bp and 322 bp) and uncleaved DNA band (456 bp) that represents unmethylated syncytin-1.

For DNA sequencing, the PCR-amplified DNA fragments were purified with PCR purification Kit (Qiagen, Valencia, CA, USA) and subcloned into the pCR2.1 TA cloning vector (Invitrogen, Carlsbad, CA, USA). Under blue and white screening, white colonies were picked to grow overnight culture in Luria–Bertani (LB) Broth medium. Plasmid DNA was purified with QIAprep Spin Miniprep Kit (Qiagen, Valencia, CA, USA) and positive clones were confirmed following restriction digestion with EcoR I and gel electrophoresis. Three positive colonies from each specimen, and 4 specimens for each of normal and carcinoma groups, were sequenced (ACGT, Inc., Wheeling, IL, USA). The methylation status of the seven CpG dinucleotides including the one used for COBRA was analyzed individually as well as in a combined manner.

### Statistical analysis

Data were analyzed using the SPSS 17.0 statistics package (SPSS, Chicago, IL, USA). Clinical information was presented as the frequencies or Mean ± SEM. Student’s *t* test was used to compare the quantitative data. Spearman’s correlation analysis method was applied to determine the correlations of IHC intensity scores with clinical grade, stage, or age. Mantel-Haenszel chi-square test (Linear-by-Linear Association) was used to analyze the correlation between IHC intensity scores and gender. The correlation between DNA methylation and gene expression were evaluated using Spearman’s correlation analysis. p value ≤ 0.05 was considered statistically significant.

## Results

### Decreased syncytin-1 mRNA levels in pancreatic cancer tissues

**Fig 1:**
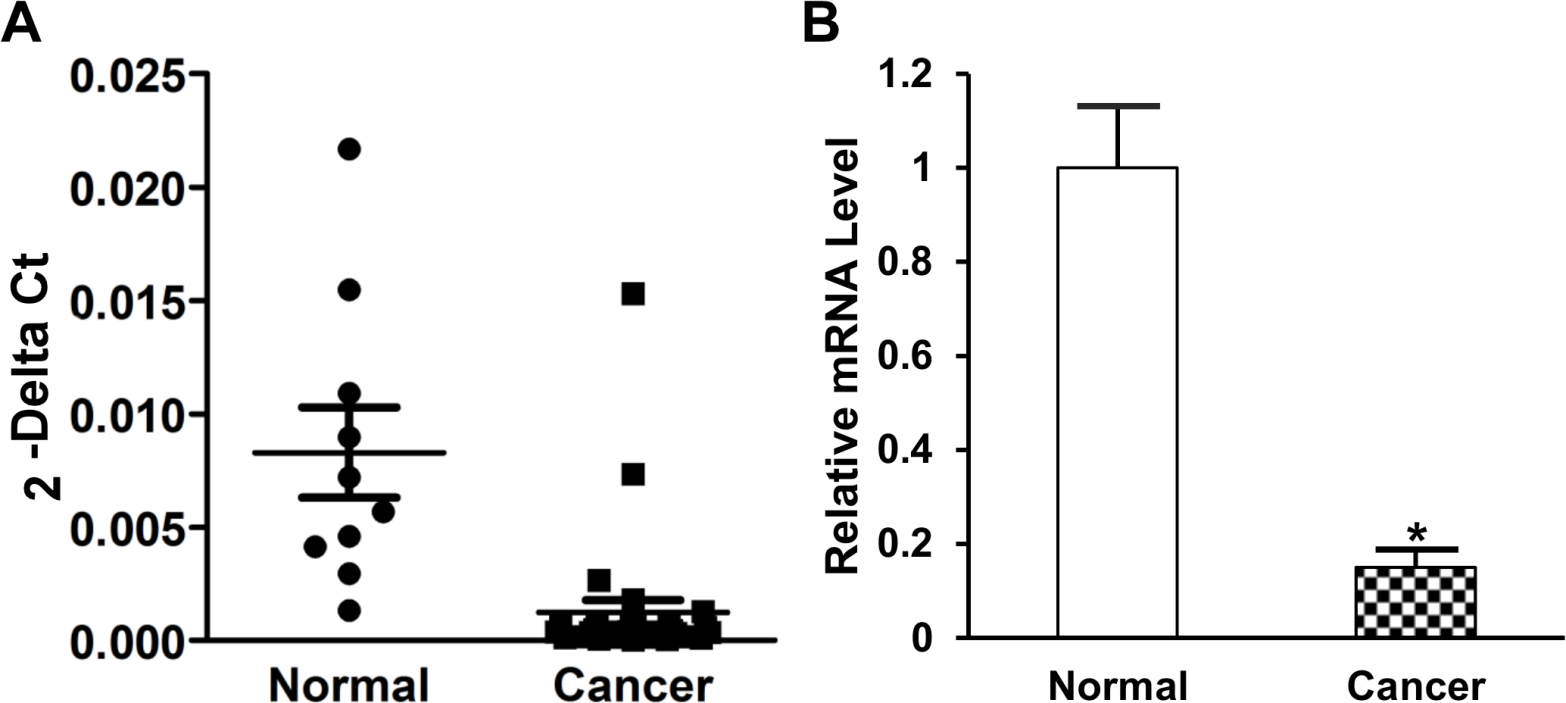
Syncytin-1 mRNA expression levels in normal pancreas and pancreatic cancer tissues. The relative syncytin-1 mRNA expression levels in normal pancreatic tissues adjacent to cancer lesions (n = 10) and pancreatic adenocarcinoma tissues (n = 30) were determined by real-time PCR. Data were standardized by the results from correspondent β-actin internal control. A. The scatter plots represented the 2^−ΔCt^ value of each sample in both the normal and cancer groups. B. The mRNA level of normal pancreas group was set as 1. Data are shown as Mean ± SEM. *: P < 0.05, as determined with Student’s *t* test.

Despite the previous failure in other laboratories to detect syncyntin-1 mRNA in pancreatic tissues using Northern hybridization technique, applying the more sensitive real-time PCR techniques, we were able to observe syncytin-1 mRNA expression in the normal pancreatic tissues adjacent to cancer lesions. Interestingly, results from real-time PCR indicated a significantly lower level of syncytin-1 mRNA in pancreatic adenocarcinoma compared to normal pancreatic tissues adjacent to cancer lesions (p<0.001) (Fig 1).

### Decreased syncytin-1 protein expression in pancreatic cancer tissues

**Fig 2:**
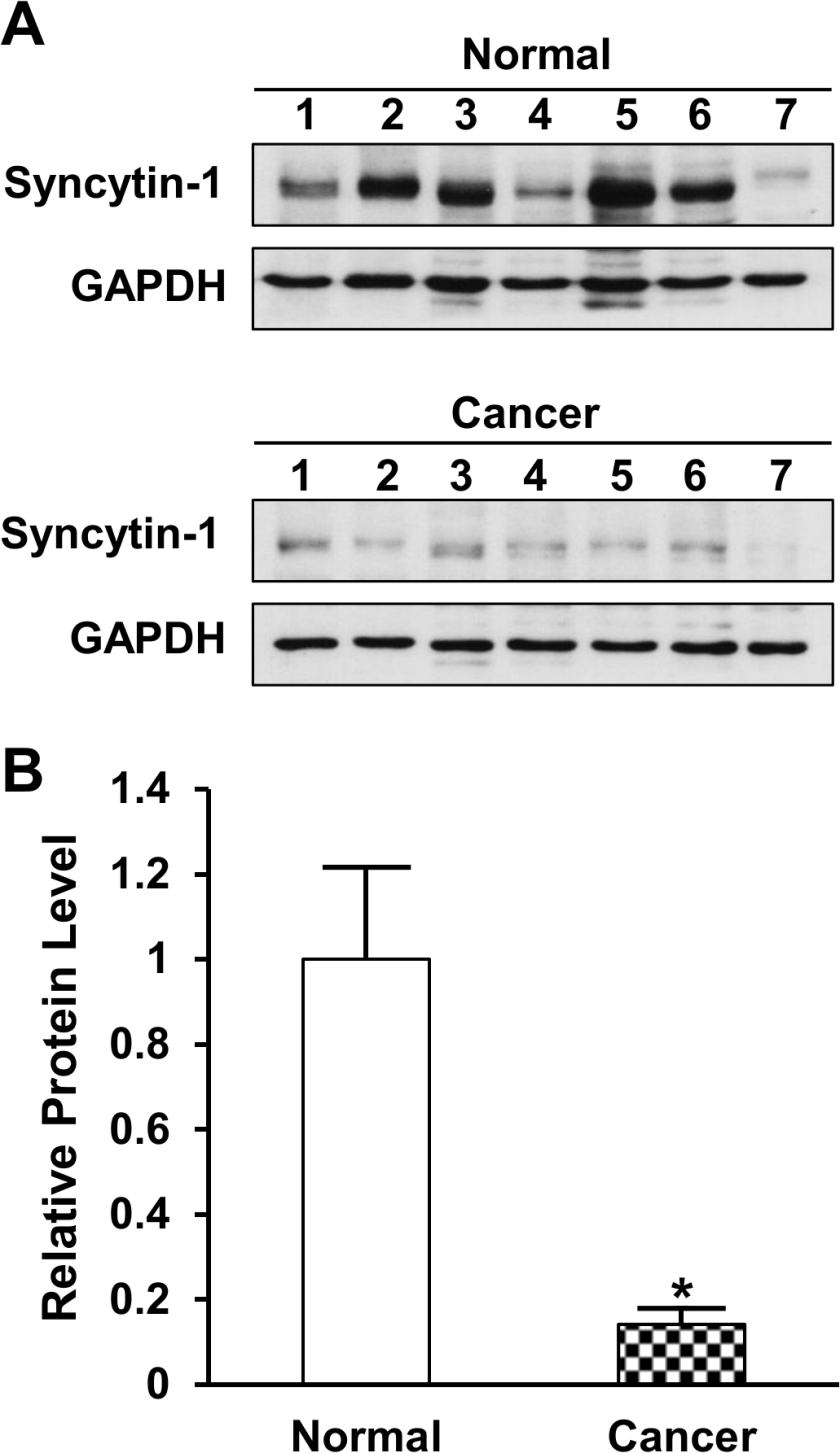
Protein levels of syncytin-1 in pancreatic tissues. A. The protein levels of syncytin-1 and GAPDH were determined with Western blot using rabbit anti-syncytin-1 polyclonal antibody or rabbit anti-GAPDH polyclonal antibody, respectively. Results of GAPDH served as protein loading controls. Normal pancreatic tissues adjacent to carcinomas (n = 7) and pancreatic adenocarcinoma tissues (n = 7) were subject to protein extraction and Western blot analyses. The experiment was performed three times and representative results were shown. B. Results of densitometry analyses indicated decreased syncytin-1 protein expression in pancreatic adenocarcinoma in comparison to normal pancreatic tissues adjacent to carcinomas. The syncytin-1 expression data were standardized by those from correspondent GAPDH bands. Data are shown as Mean ± SEM. *: P < 0.05, as determined with Student’s *t* test.

To further confirm the syncytin-1 expression in pancreas tissues, we performed Western blot analysis on proteins extracted from frozen cancer-adjacent normal tissues and pancreatic adenocarcinoma tissues. Using a syncytin-1-specific antibody, a distinct 55 kDa band representing syncytin-1 was detected in normal pancreas tissues. Fig 2A upper panel is a representative image from repeated experiments. Densitometry analysis of the results indicated that, consistent with the mRNA levels, pancreatic carcinoma tissues expressed decreased syncytin-1 protein compared to cancer-adjacent normal tissues (Fig 2B).

### Immunohistochemistry analysis of pancreatic cancer tissue microarray

**Fig 3:**
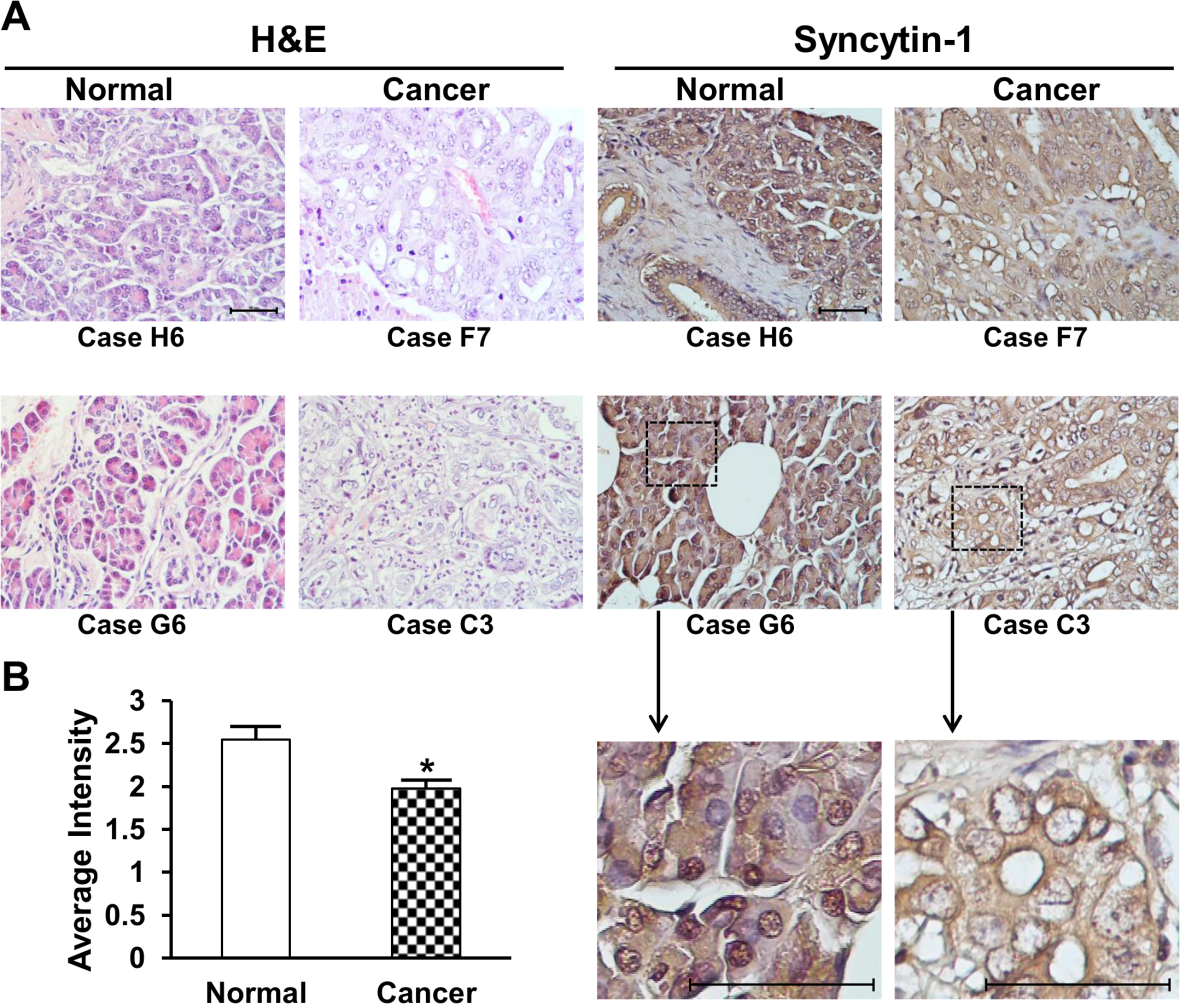
Immunohistochemistry analyses of syncytin-1 expression in pancreas tissues. The pancreatic adenocarcinoma tissue array was processed as described under Materials and Methods. A. Left panels are representative images showing the results of H&E staining of one normal pancreatic tissue (Case H6), one normal pancreatic tissue adjacent to adenocarcinomas (Case G6), and two adenocarcinoma tissues (Case F7 and C3). Labels below the images indicate tissue positions in the array. Right panels are IHC results for syncyin-1 expression in pancreatic tissues, in the same order as in H&E staining. The original magnification is 40×10. Scale bars indicate 50 μm. Dashed lines in Case G6 and C3 indicated the areas zoomed-in for 4 folds. Syncytin-1 protein was stained in yellow to brown color. Cell nuclei were counterstained in blue color with haematoxylin. Syncytin-1 protein localized mostly on the membrane and in cytoplasm, with lower staining observed in nucleus, for both normal and carcinoma tissues. Reduced level of syncytin-1 expression was found in pancreatic adenocarcinomas in comparison to normal tissues. Diminished level of staining signals was observed in stromal cells. B. Syncytin-1 expression was scored based on the staining intensity for all cases in the array. Average intensities from normal group and cancer group were calculated and compared with Student’s *t* test. Data are shown as Mean ± SEM.*: P < 0.05.

To further confirm the syncytin-1 protein expression pattern in benign and malignant pancreas tissues, we performed immunohistochemistry assay using a tissue microarray containing 10 cores of normal pancreas from non-cancer patients, 10 cores of normal tissues adjacent to pancreatic cancer and 60 cores of pancreatic adenocarcinomas. The demographic information of pancreatic cancer patients was documented on the website of the manufacturer (http://www.biomax.us/tissue-arrays/Pancreas/PA805). Generally, normal pancreas tissues from non-cancer patients and normal tissues adjacent to pancreatic cancers displayed comparable density of syncytin-1 staining signals. However, lower staining densities were observed in pancreatic cancer tissues than the two normal groups (Fig 3). This result was consistent with those from mRNA measurement and Western blot analysis shown above (Figs 1 and 2), confirming the decreased syncytin-1 expression in pancreatic cancers. Close inspection on the distribution of staining signals indicated that syncytin-1 broadly distributed on cell membrane, cytoplasm and nucleus of glandular epithelial cells (Fig 3). Little or no syncytin-1 expression was found in stromal cells. Further analyses showed that syncytin-1 expression levels were negatively correlated with patients’ age (n = 51, r = -0.300, p = 0.032), but not correlated with cancer stage, grade, invasion or gender (P>0.05) (Table 1).

**Table 1:** Correlation analyses between syncytin-1 expression and clinical parameters.

Characteristics	Case numbers	Scores^a^			Spearman r	P value
		Score = 1	Score = 2	Score = 3		
**Grade**					-0.066	0.648
Grade 1	6	0	4	2		
Grade 2	20	4	14	2		
Grade 3	24	5	14	5		
**Stage**					0.195	0.170
Stage I	13	3	9	1		
Stage II	28	5	16	7		
Stage III+IV	10	1	6	3		
**Invasion**					0.160	0.261
T1+T2	18	3	14	1		
T3+T4	33	6	18	9		
**Age**					-0.300	0.032*
Age≤50	13	2	6	5		
50<Age≤60	20	3	12	5		
60<Age≤80	18	4	14	0		
**Gender**					χ^2^ = 0.068^b^	0.794
Male	29	4	21	4		
Female	22	5	11	6		

^a^: Score = 0 was excluded because all cases were expressed syncytin-1.

^b^: Using Mantel-Haenszel chi-square test (Linear-by-Linear Association) to analyze.

*: P<0.05.

### Decreased syncytin-1 expression in pancreatic cancer is associated with DNA hypermethylation

**Fig 4:**
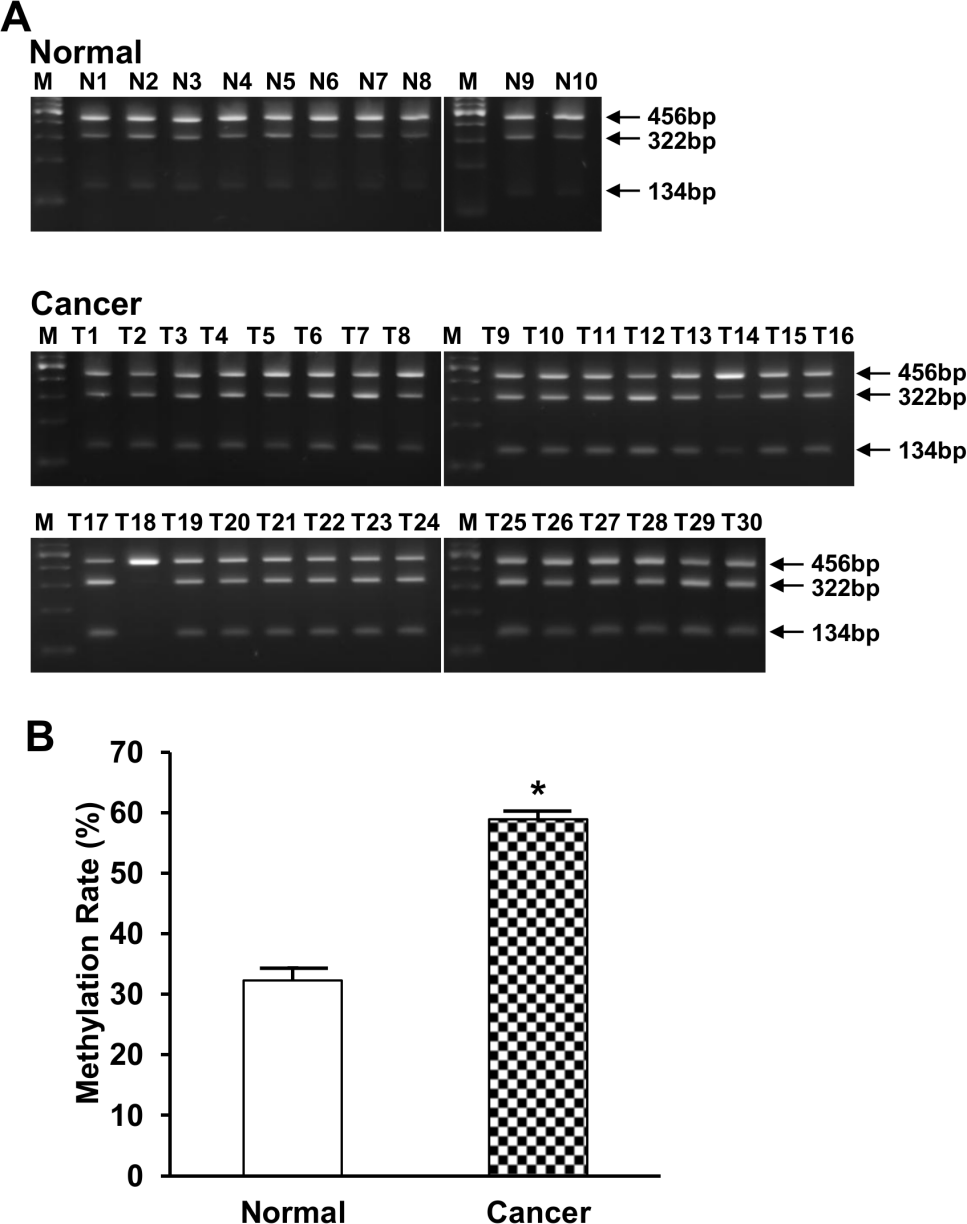
DNA methylation of syncytin-1 gene measured by COBRA. A. Using bisulfite-treated genomic DNA as template, PCR amplicon representing a 456 bp of the 5’ LTR promoter region of syncytin-1 gene were treated with an excess of restriction enzyme Acl I. The digested PCR products were separated in 2.0% agarose gel electrophoresis and DNA bands were visualized by ethidium bromide staining. The 134 bp and 322 bp cleavage products represent methylated syncytin-1 promoter, while the 456 bp band represents the unmethylated syncytin-1 promoter. B. The methylation index was calculated by densitometry analyses of the electrophoresis images. COBRA results indicated an increased methylation in pancreatic adenocarcinoma compared to normal pancreatic tissues. Data were presented as Mean ± SEM.*: P < 0.05, as determined with Student’s *t* test.

**Fig 5:**
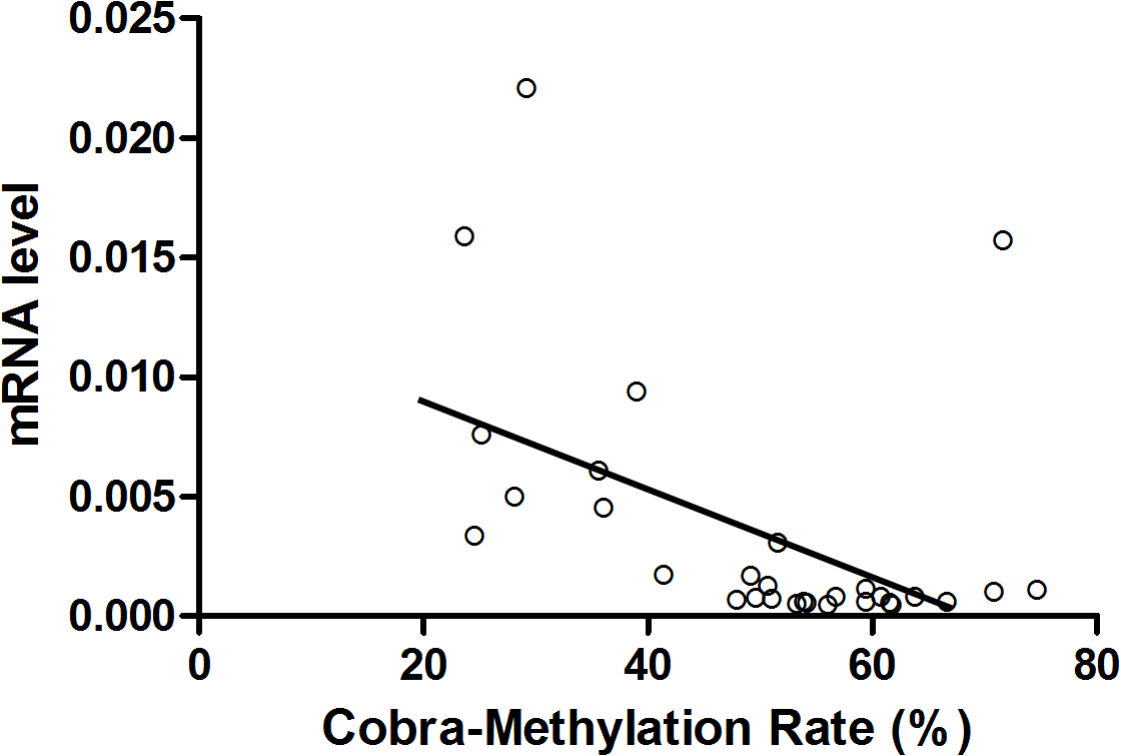
Correlation between syncytin-1 expression and syncytin-1 methylation index in normal and adenocarcinoma tissues. The Y-axis indicated syncytin-1 mRNA levels and the X-axis represented syncytin-1 DNA methylation index. Spearman’s correlation analysis showed a highly significant positive correlation between syncytin-1 mRNA levels and syncytin-1 promoter methylation levels among normal and adenocarcinoma tissues (r = -0.558, p = 0.001).

Previous studies have shown that syncytin-1 gene expression in placenta was dynamically regulated under different pathophysiological conditions such as hypoxia and preeclampsia [36, 37]. DNA methylation of the 5’ LTR of syncytin-1 gene appears to not only define its tissue-specific expression pattern, but also be implicated in the temporal regulation of syncytin-1 expression during placental maturation along the pregnancy [29, 30, 38]. We performed COBRA to assess DNA methylation levels in the 5’ LTR of syncytin-1 gene in relatively normal pancreatic tissues adjacent to cancer and cancer tissues. As shown in Fig 4A, methylated syncytin-1 gene was digested by Acl I to generate 2 fragments of 134 bp and 322 bp, whereas the unmethylated counterpart could not be cleaved and sustained the size of 456 bp. Densitometry analysis indicated that pancreatic adenocarcinoma (n = 30) had significantly higher methylation levels than the normal tissues adjacent to cancer tissues (n = 10, p<0.001) (Fig 4B). Moreover, the DNA methylation index was inversely correlated with the correspondent mRNA levels (r = -0.558, p = 0.001) (Fig 5). Thus, both the comparison by groups (Cancer group with lower average syncytin-1 mRNA level had higher average methylation index) and correlation analysis by individual samples pointed to an inhibitory effect of hypermethylation on the expression of syncytin-1 gene in pancreatic cancer tissues.

### Bisulfite sequencing of the syncytin-1 5’ LTR

**Fig 6:**
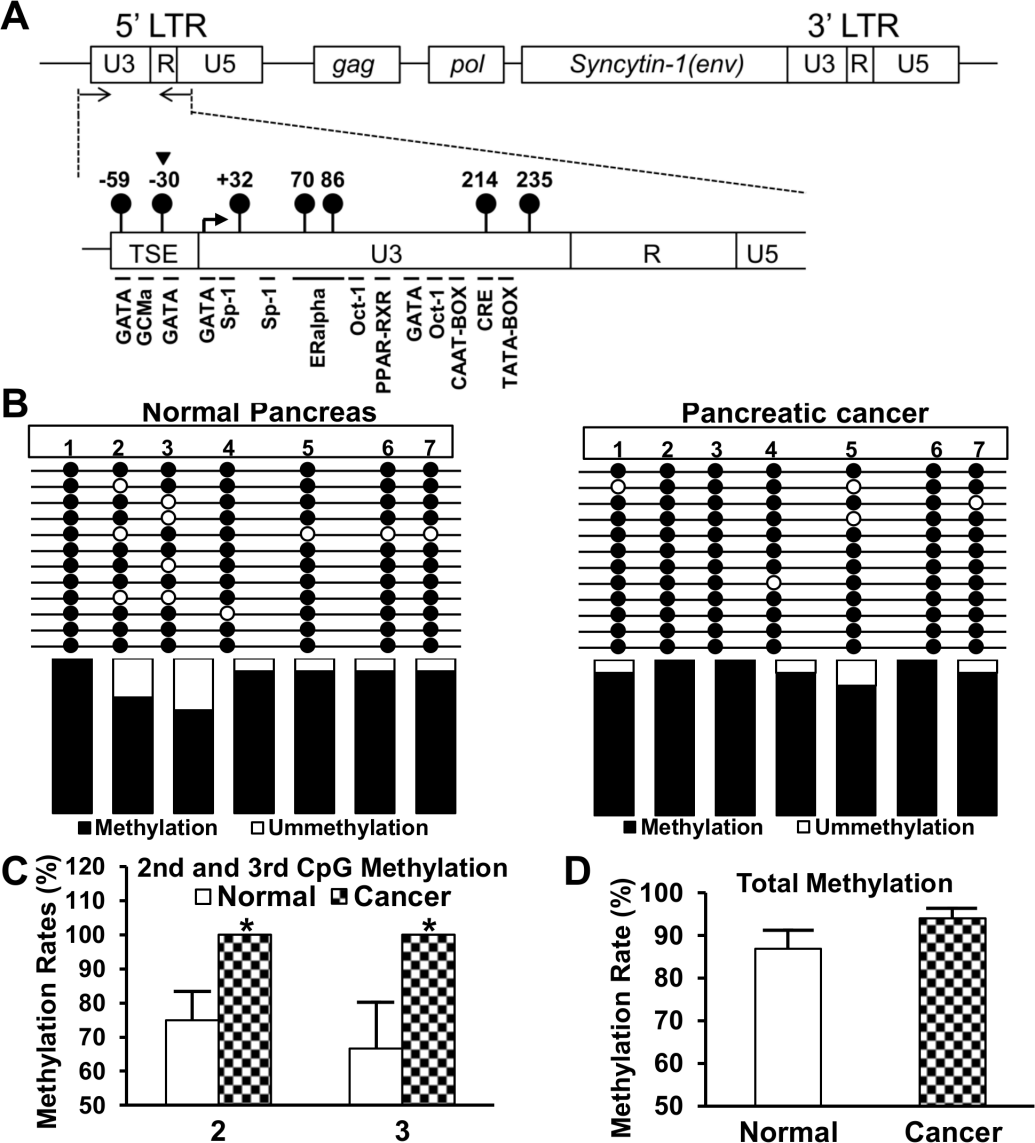
Bisulfite sequencing of the syncytin-1 gene 5’ LTR promoter region. Bisulfite-converted DNA from pancreatic normal (n = 4) and carcinoma (n = 4) tissues were PCR amplified, subcloned, and sequenced. A. Upper panel: Schematic map of HERV-W gene. Arrows show the location of PCR primers. Lower panel: The solid lollipops indicate the seven CpG sites in the PCR fragments and the numbers indicate the CpG positions. A triangle points to the second CpG site that was used for COBRA. TSE, a trophoblast-specific enhancer region, is directly upstream of 5’ LTR U3 region. Important cis-elements are highlighted below the PCR fragment. The angled arrow indicates the transcription start site. B. The sequencing result of syncytin-1 gene 5’ LTR promoter region. The solid and open circles represent the methylated or unmethylated cytosines, respectively, in CpGs dinucleotide contexts. The average methylation levels for each CpG site were shown in columns below. C. and D. Quantitative comparison of the syncytin-1 DNA methylation levels between pancreatic normal and carcinoma tissues. C. There was a significant increased methylation in the second and third CpG sites in pancreatic carcinomas than normal tissues. D. DNA methylation index of seven CpG sites showed a trend for increased methylation in pancreatic carcinomas compared to normal tissues, but the difference did not reach a statistical significance (P > 0.05). Data are expressed as Mean ± SEM. *: P < 0.05, as determined with Student’s *t* test.

Bisulfite sequencing was performed as a confirmatory measure for characterization of the syncytin-1 gene methylation patterns. We determined the DNA site-specific methylation status in the cancer tissues and normal tissues adjacent to cancer lesions. As shown in Fig 6A, the sequenced area harbors seven CpG and multiple transcription factor binding sites. Among the seven CpG dinucleotides examined, the second and third CpG sites had dramatically altered DNA methylation levels, with both sites showing complete methylation in pancreatic cancers, but a lower methylation levels (75.0%, p = 0.024; 66.7%, p = 0.049) in cancer-adjacent normal tissues (Fig 6B and 6C). The second CpG dinucleotides is located in the restriction site used for COBRA (Fig 6A), and the consistent results from two independent assays validated the accuracy of these experiments. The methylation levels of the remaining sites were largely unchanged (Fig 6B). When results from all sites were pooled together, the cancer group showed a higher methylation levels than the cancer-adjacent normal group, but the difference did not reach a statistical significance level (Fig 6D) (p = 0.18). Thus, syncytin-1 hypermethylation alterations displayed a site-specific, rather than an “all out” pattern, in pancreatic cancer tissues.

## Discussion

Among the *env* genes of HERVs, only HERV-E gene was known to express in the human pancreas [39]. Yi et al. reported that the HERV-W gene encoding syncytin-1 was expressed in the pancreas of Japanese monkey [34]. But a study using Northern hybridization analysis failed to detect syncytin-1 gene expression in human pancreas [11]. Here we demonstrated that syncytin-1 is expressed at moderate-to-high levels in human pancreas on both mRNA and protein levels based on the consistent results of real-time PCR, Western blot and immunohistochemistry (Figs 1–3). To our knowledge, this is the first report on the presence of syncytin-1 mRNA and protein expression in human normal pancreas and pancreatic cancer tissues.

Syncytin-1 was known to mediate the fusion of mononucleated cytotrophoblasts into multinucleated syncytiotrophoblasts during the maturation of placenta [11, 14, 15]. New evidence indicated that syncytin-1 is a multifunctional protein. In addition to fusogenic activity, syncytin-1 is involved in immune suppression and may contribute to the maternal adaptation to pregnancy [40–42]. Huang et al. reported that syncytin-1 is able to promote G1/S transition via regulating CDK4, E2F1, PCNA, c-Myc and p15 expression in placental choriocarcinoma BeWo and Chinese hamster ovary (CHO) cells [22]. Moreover, placental trophoblast survival appears to rely on a sufficient level of syncytin-1, since knockdown of syncytin-1 expression induced extensive cell apoptosis [20]. Both the fusogenic and nonfusogenic activities have been used to explain the function and homeostasis of trophoblast lineage. Stress-induced upregulation of syncytin-1 expression may promote the growth of cytotrophoblasts, at the same time, generate more syncytiotrophoblasts through the fusogenic and anti-apoptotic activities. Under pathological conditions, decreased syncytin-1 expression may lead to cell death and trophoblast deportation, a phenomenon frequently observed in preeclamptic placentas [20, 23]. These findings raised a question on the function of syncytin-1 in human pancreas. From the immunostaining image, syncytin-1 appeared to be restricted to glandular epithelial cells (Fig 3A). Its broad distribution in cell membrane, cytoplasm, and nuclei may suggest multiple functions in human pancreas. Since cell fusion has not been observed in pancreatic cells, the nonfusogenic activity is most likely involved in any role(s) that syncytin-1 may potentially play in human pancreas biology.

Syncytin-1 overexpression has been found in a variety of human malignancies including breast cancer, endometrial carcinoma, ovarian cancer, colorectal cancer, leukemia and lymphoma [16–19, 26, 28]. Larsson et al. reported that 38% of breast cancers expressed syncytin-1 and the syncytin-1 expression levels positively correlated with benign prognosis for longer recurrence-free survival [16]. Strissel et al. found that syncytin-1 was overexpressed in advanced stage and less differentiated endometrial carcinomas comparing to early stage and well differentiated carcinomas [18]. Larsen et al. reported that increased syncytin-1 expression was associated with decreased overall survival in rectal cancer but not in colonic cancer [19]. High expression of syncytin-1 has been also detected in human endometriotic tissues, the ectopic overgrowth of endometrium, which may share some common features with endometrial cancers [35]. Thus, the clinical implications of syncytin-1 expression level seem to vary among different tumor types and stages. Regardless the complicated relationship between syncytin-1 expression and clinical manifestations, syncytin-1 overexpression as comparison to correspondent normal tissues appears to be feature frequent even in human malignances. Consequently, the growth-promoting and anti-apoptotic activities of syncytin-1 may be cited to explain its overexpression in malignant cells [20, 22]. By this consideration, it is a surprise to see a reduced syncytin-1 expression in pancreatic cancer cells when compared to normal tissues from non-cancer patients as well as normal tissues adjacent to cancer lesions (Figs 1–3). This exceptional expression pattern seemed to suggest a distinct biological function of syncytin-1 in pancreatic cancers. Limited by the lack of clinical data we could not investigate the relationship between syncytin-1 expression levels and patient survival. Nevertheless, we were able to analyze the association with age, gender, histological grade and clinical stage (Table 1). An inverse correlation between syncytin-1 expression levels and age was observed. Given the relatively small sample size, the possible role of syncytin-1 in the pathogenesis and progression of pancreatic cancers could not be excluded. More studies in a larger cohort are required to verify the findings on this unique expression pattern and to delineate its function(s) in pancreatic cancers.

Despite the intriguing expression pattern of syncytin-1 in the current study opposite to previous observations in other human malignancies, the relationship between syncytin-1 gene methylation and expression appear to be a reminiscent of the repeatedly observed pattern, that is, a close association of higher expression with the hypomethylated 5’ LTR. Previous studies showed that preeclamptic placenta had decreased syncytin-1 expression and hypermethylated 5’ LTR [43]. In endometrial cancers, the hypomethylated 5’ LTR was associated with the upregulated expression of syncytin-1 [18]. The same inverse correlation between the 5’ LTR methylation and expression levels was found in this study on normal and cancerous pancreas tissues (Fig 5). It is noteworthy that bisulfite sequencing results indicated that two specific CpG sites, immediately upstream and downstream of the transcription start site (Fig 6A), may play critical roles in the regulation of the syncytin-1 expression. Indeed, we previously observed that the second CpG was hypermethylated in normal endometrium, but became hypomethylated in endometriotic tissues, leading to the overexpression of syncytin-1 gene [35]. In another study, we detected significant hypermethylation of the third CpG and a decreased syncytin-1 gene expression in preeclamptic placentas [43]. Studies from other groups also detected alterations of DNA methylation levels in the third CpG site [29, 30]. Bisulfite sequencing results of Matouskova et al. showed 45% methylation at this site in placenta, and 100% in skin fibroblasts [29]. Putting together, these data highlighted the significance of these two CpG sites in the control of syncytin-1 gene expression. It should be pointed out that transcriptional factor GCMa, a placenta-specific transcriptional factor, binds to two sites in 5’ LTR of syncytin-1 and transactivates syncytin-1 gene expression in trophoblast cells [44]. One GCMa binding site is located in a 33-bp trophoblast-specific enhancer (TSE) region, which is directly upstream of the syncytin-1 gene 5’ LTR (Fig 6A) [45, 46]. Interestingly, the second CpG site is closely located, only 4 nucleotides downstream of the GCMa binding site in the TSE region (Fig 6A) [45]. The third CpG site falls right in the middle between Pit-1a and Sp-1 transcription factor binding sites (Fig 6A) [30]. It has been reported that DNA methylation, besides its effect on histone code modification, alters the charge and structure of DNA duplex, affects the interaction between DNA and transcriptional factors [47–49], and ultimately, modulates gene transcription. Identification of the two key CpG sites has provided valuable information for the design of in-depth studies to delineate the epigenetic and non-epigenetic mechanisms regulating syncytin-1 expression under different pathological conditions.

## Conclusions

This study revealed a constitutive expression of syncytin-1 in normal human pancreas. Decreased expression of syncytin-1 and significantly hypermethylated CpG dinucleotides at the 5’ LTR of syncytin-1 gene were found in pancreatic adenocarcinomas. Methylation status of two CpG sites from this region that harbors key transcriptional factors is closely associated with the expression of syncytin-1 gene. These findings have shed new lights on the epigenetic regulation of syncytin-1 in pancreatic cancers. The pathophysiological function of syncytin-1 and the exact molecular mechanisms mediating the epigenetic regulation of syncytin-1 in pancreatic cancer remain to be investigated.

## Supporting Information

S1 Fig The original Western blot results of normal and cancer samples for GAPDH. Blot M18 is a group of pancreatic cancer samples, and blot M19 is normal pancreas tissues adjacent to cancer lesions. Blots M18 and M19 were used to detect GAPDH protein expression levels as internal controls using rabbit anti-GAPDH monoclonal antibody. The size of target protein, GAPDH is approximately 37 kDa.
(TIF)

S2 Fig The original Western blot detecting syncytin-1 protein expression in normal and cancer samples. After detection of GAPDH the blots were striped, and blocked in TBST containing 5% non-fat milk. The blots were incubated with rabbit polyclonal anti-syncytin-1 antibody from Gene Tex (Cat# GTX70327). Due to incomplete membrane stripping and the same species for primary GAPDH and syncytin-1 antibodies, GAPDH bands were also shown when syncytin-1 was detected, as pointed by an arrows in the image. The size of target protein, syncytin-1, is approximately 55 kDa.
(TIF)

S1 File DNA sequence for COBRA and methylation sequencing. (PDF)

S1 Table The clinical characters of pancreatic cancer patient subjects. (XLSX)

S2 Table The raw data of 2^−ΔCt^ values from real-time PCR. (XLSX)

S3 Table The raw data of syncytin-1 Western blot bands density. (XLSX)

S4 Table Clinical data of tissue array and IHC density scores on syncytin-1 expression. (XLSX)

S5 Table The raw data of band density from COBRA. (XLSX)

S6 Table The raw data of methylation sequencing. (XLSX)
