# Supplementary figures and images for: Promoter Hypermethylation and Decreased Expression of Syncytin-1 in Pancreatic Adenocarcinomas
Source: PLoS One. 2015 Jul 31;10(7):e0134412. doi: 10.1371/journal.pone.0134412 (PMC4521816; doi:10.1371/journal.pone.0134412)

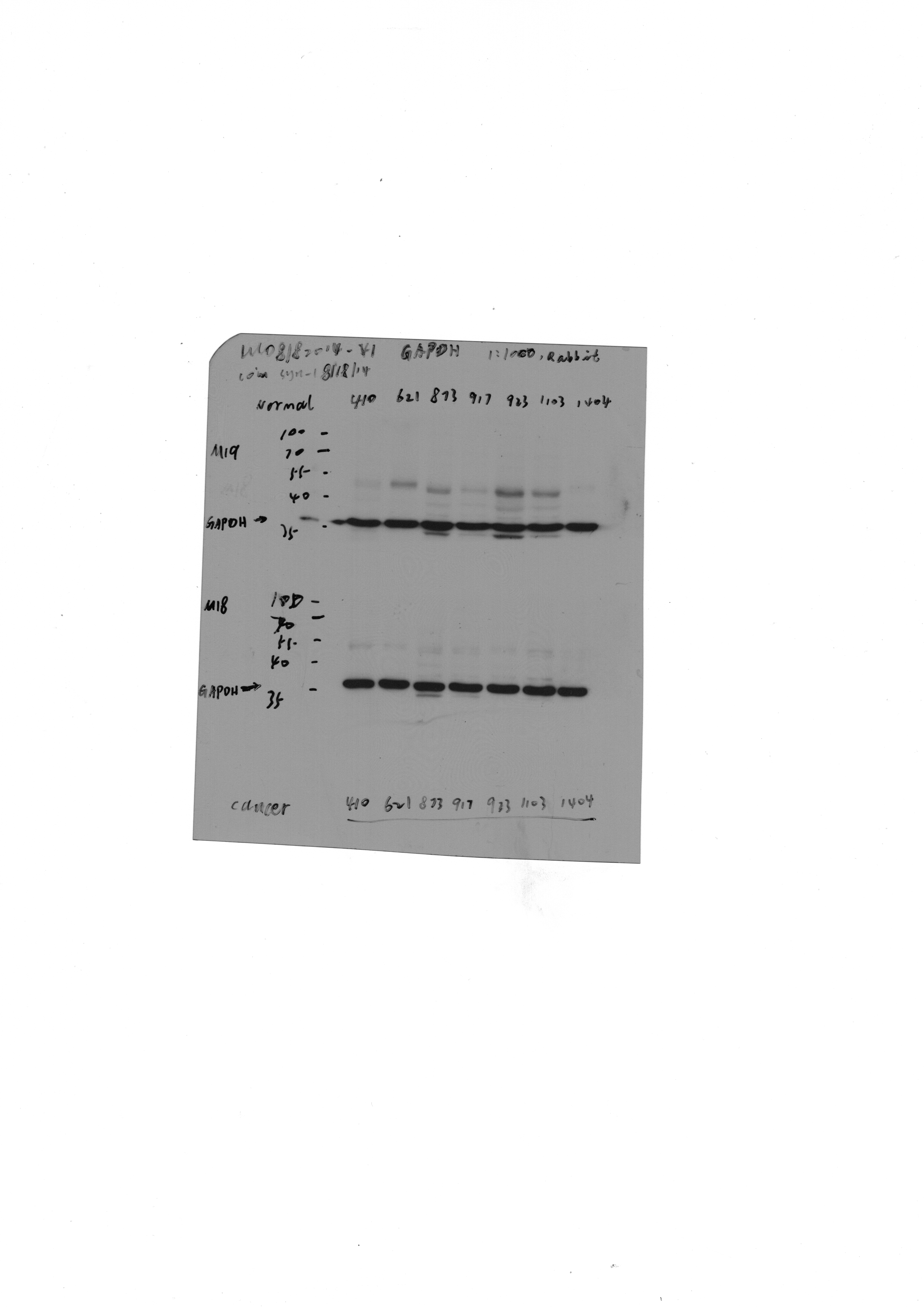

Supplement: S1 Fig — Blot M18 is a group of pancreatic cancer samples, and blot M19 is normal pancreas tissues adjacent to cancer lesions. Blots M18 and M19 were used to detect GAPDH protein expression levels as internal controls using rabbit anti-GAPDH monoclonal antibody. The size of target protein, GAPDH is approximately 37 kDa. (TIF) [file pone.0134412.s001.tif]

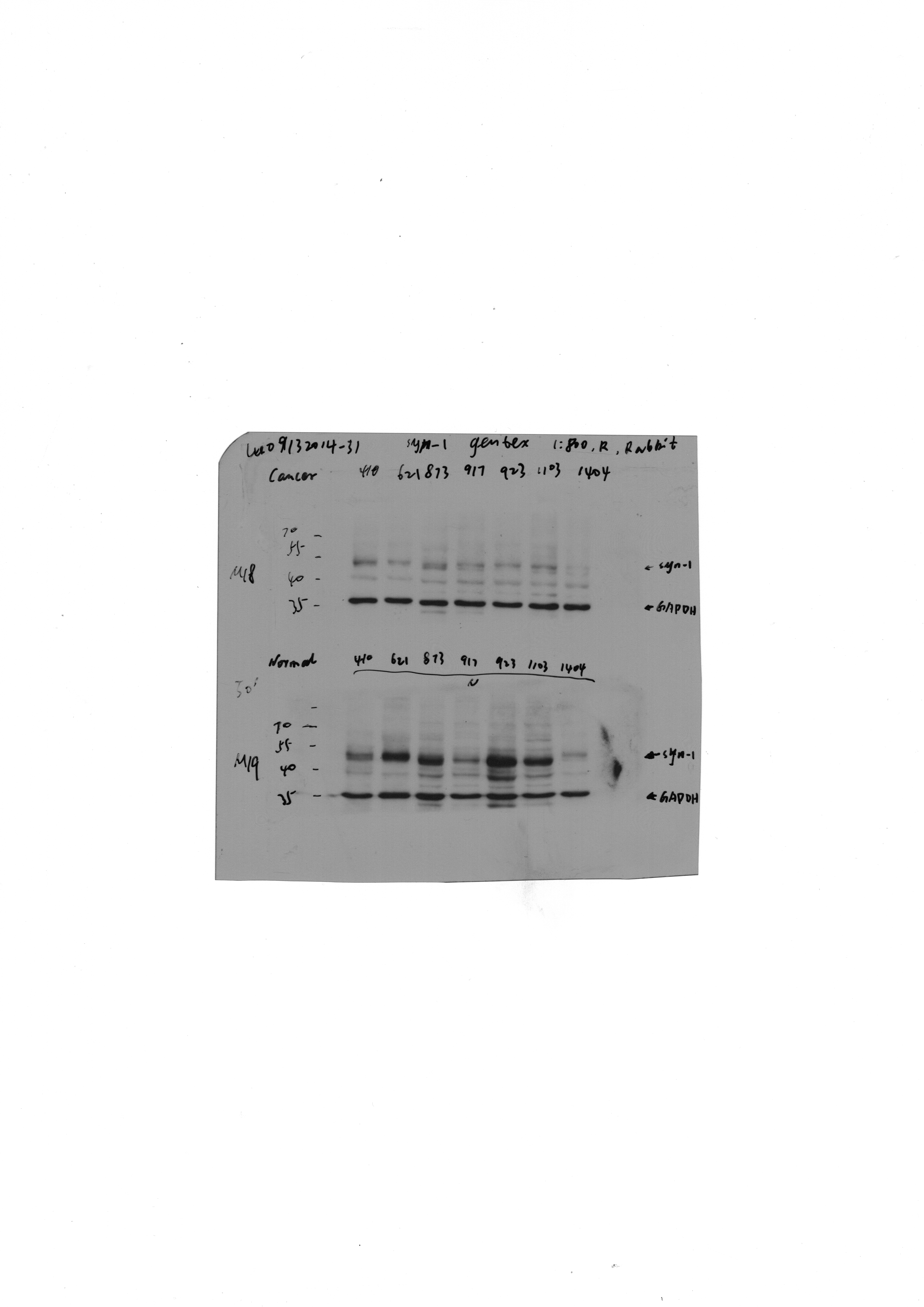

Supplement: S2 Fig — After detection of GAPDH the blots were striped, and blocked in TBST containing 5% non-fat milk. The blots were incubated with rabbit polyclonal anti-syncytin-1 antibody from Gene Tex (Cat# GTX70327). Due to incomplete membrane stripping and the same species for primary GAPDH and syncytin-1 antibodies, GAPDH bands were also shown when syncytin-1 was detected, as pointed by an arrows in the image. The size of target protein, syncytin-1, is approximately 55 kDa. (TIF) [file pone.0134412.s002.tif]
