# Supplementary material for: Promoter Hypermethylation and Decreased Expression of Syncytin-1 in Pancreatic Adenocarcinomas
Source: PLoS One. 2015 Jul 31;10(7):e0134412. doi: 10.1371/journal.pone.0134412 (PMC4521816; doi:10.1371/journal.pone.0134412)
Supplement: S1 File — (PDF) [file pone.0134412.s003.pdf]

Original DNA sequence for Cobra and Sequence Assay (5’-3’)

ATACTCTCTGGAGAGTGAATTACTGAGTCACATGATCTTCACTGCAGTCA
TTTGTGGCTATGTGACATAGTTCTGGACAGTGAACATAGACAGAAGTCCCTGGGGCGGGC
TTCCTTTCTGGGATGAGGGCAA***AACGCC***TGGAGATACAGCAATTATCTTGCAACTGAGAG
ACAGGACTAGCTGGATTTCCTAGGCCGACTAAGAATCCCTAAGCCTAGCTGGGAAGGTGA
CCACGTCCACCTTTAAACACGGGGCTTGCAACTTAGCTCACACCTGACCAATCAGAGAGC
TCACTAAAATGCTAATTAGGCAAAGACAGGAGGTAAAGAAATAGCCAATCATCTATTGCC
TGAGAGCACAGCAGGAGGGACAACAATCGGGATATAAACCCAGGCATTCGAGCTGGCAAC
AGCAGCCCCCCTTTGGGTCCCTTCCCTTTGTATGGGAGCTGTTTTC

Convert all C to T

ATATTTTTTGGAGAGTGAATTATTGAGTTATATGATTTTTATTGTAGTTA
TTTGTGGTTATGTGATATAGTTTTGGATAGTGAATATAGATAGAAGTTTTTGGGGTGGGT
TTTTTTTTTGGGATGAGGGTAAAATGTTTGGAGATATAGTAATTATTTTGTAATTGAGAG
ATAGGATTAGTTGGATTTTTTAGGTTGATTAAGAATTTTTAAGTTTAGTTGGGAAGGTGA
TTATGTTTATTTTTAAATATGGGGTTTGTAATTTAGTTTATATTTGATTAATTAGAGAGT
TTATTAAAATGTTAATTAGGTAAAGATAGGAGGTAAAGAAATAGTTAATTATTTATTGTT
TGAGAGTATAGTAGGAGGGATAATAATTGGGATATAAATTTAGGTATTTGAGTTGGTAAT
AGTAGTTTTTTTTTGGGTTTTTTTTTTTTGTATGGGAGTTGTTTTT

Note:

1. **Underline indicates the sequences of primers binding.**
2. **Restriction enzyme Acl I cut at AACGTT, the second CpG methylation site.**
3. **7 CG sites are indicated as above in gray brackground.**
